# Dataset on elemental concentration and group identification of ancient potteries from Tamil Nadu, India

**DOI:** 10.1016/j.dib.2016.11.075

**Published:** 2016-12-01

**Authors:** A. Chandrasekaran, A. Naseerutheen, R. Ravisankar

**Affiliations:** a Department of Physics, SSN college of Engineering, Kalavakkam, Chennai 603110, Tamil Nadu, India; b Department of Physics, C.Abdul Hakeem College, Melvisharam 632509, Tamil Nadu, India; c Post Graduate and Research Department of Physics, Government Arts College, Tiruvanamalai 606603, Tamil Nadu, India

**Keywords:** Ancient potteries, Elements, EDXRF, Factor and cluster analysis

## Abstract

The dataset contains concentration of major and trace elements of ancient potteries from Tamilnadu and grouping different potteries from the statistical techniques of factor and cluster analysis (Figs. 2, 3 and 4). The major and trace elemental concentration data generated using energy dispersive X-ray fluorescence technique (EDXRF) and factor and cluster analysis data obtained using STATISTICA (10.0 version) software. The concentration of major and trace elements determines the type of clay minerals (Calcareous/Non-Calcareous and either low or high refractory) and firing atmosphere adopted by the artisans at the time of manufacture. The statistical tool examined graphically the grouping pattern of the samples in terms of chemical composition and extract information about their provenance. The compilation of this data provides a resource for the wider research community in archeology.

**Table:** 

**Specifications**	**Table**

Subject area	Earth Science, Archaeology
More specific subject area	Archeometry
Type of data	Table, Figures
How data was acquired	Energy Dispersive X-ray Fluorescence Spectrometer (EDXRF)
Data format	Raw Analysed
Experimental factors	Powdered pottery samples were dried using hot air oven and stored in desiccators until they were analysed. One gram of the fine powder sample and 0.5 g of the boric acid (H_3_BO_3_) were mixed. This mixture was thoroughly ground and pressed to into a pellet of 25 mm diameter using a hydraulic press. The prepared pellets were analysed using the EDXRF Spectrometer.
Experimental features	Determination of elemental oxide concentration of SiO_2,_ Al_2_O_3_, CaO, Fe_2_O_3_,K_2_O, TiO_2_ and Cu, Zn, Pb, La, Co, V, Cd and Cr of ancient potteries
Data source location	Arcot, Vellore District, Tamil Nadu, India
Data accessibility	Data is with this article

**Value of the data**

• Data could be used to identify the nature of clay and raw materials to production of potteries.
• Data given here could motivate the studies on ancient artifacts in future.
• Data on factor and cluster analysis provides the grouping of ancient potteries.
• The data could be more informative to researchers investigating geographical origin and ancient artifacts from the study area.

## 1 Data

**Fig. 1:**
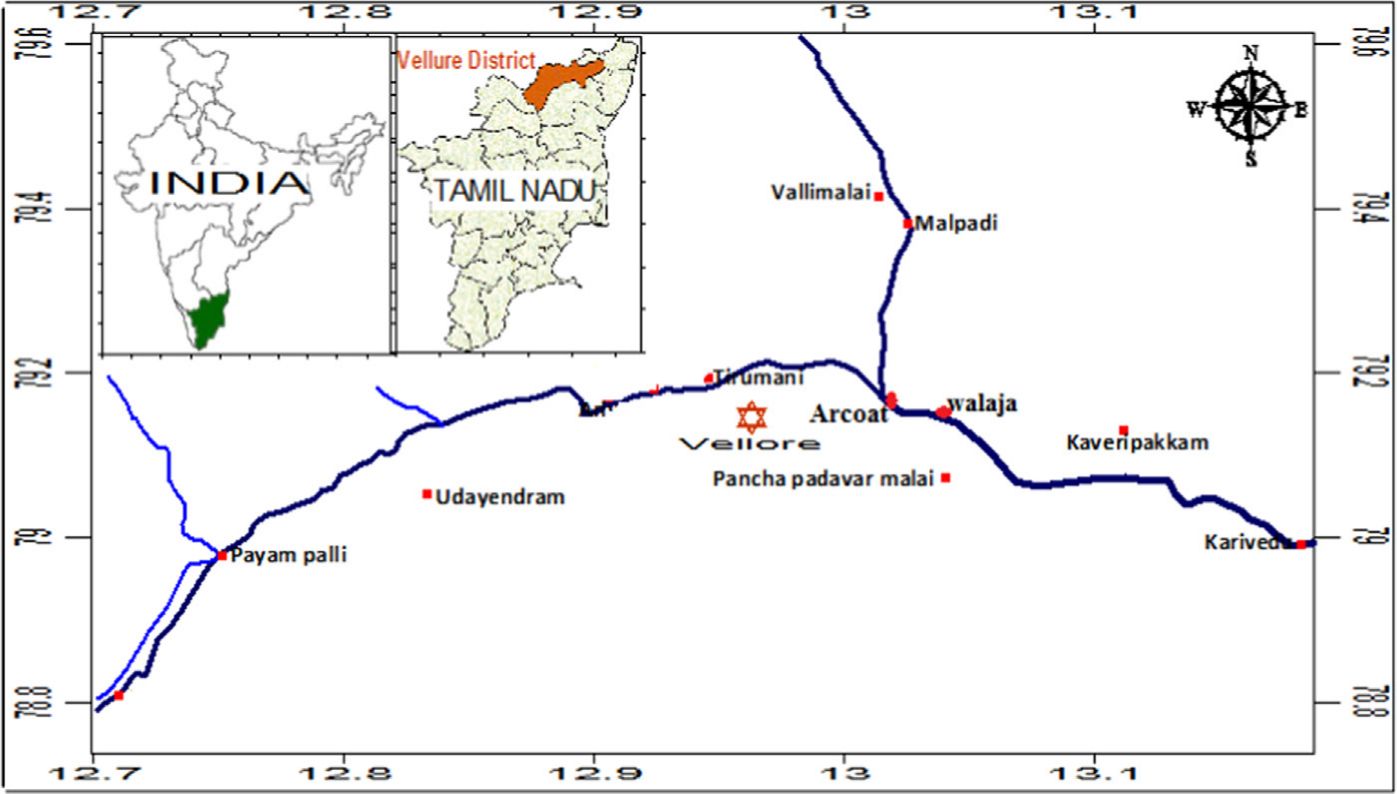
Shows the archeological sites in Arcoat , Vellore district, Tamil Nadu.

**Fig. 2:**
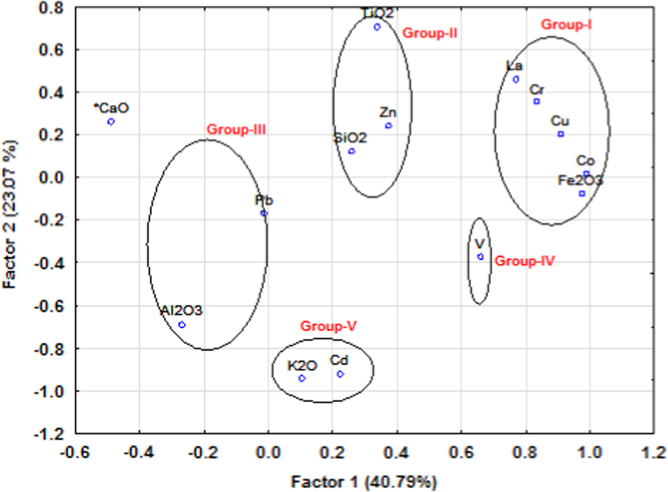
Factor score 1 Vs Score 2 of elementental concentrations.

**Fig. 3:**
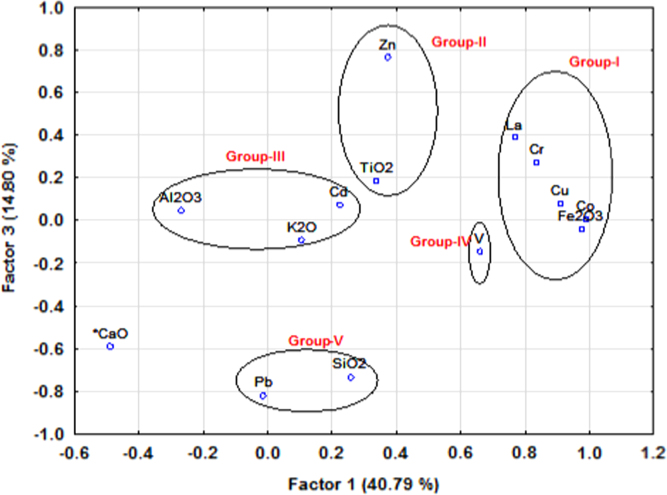
Factor score 1 Vs score 3 of elementental concentrations.

**Fig. 4:**
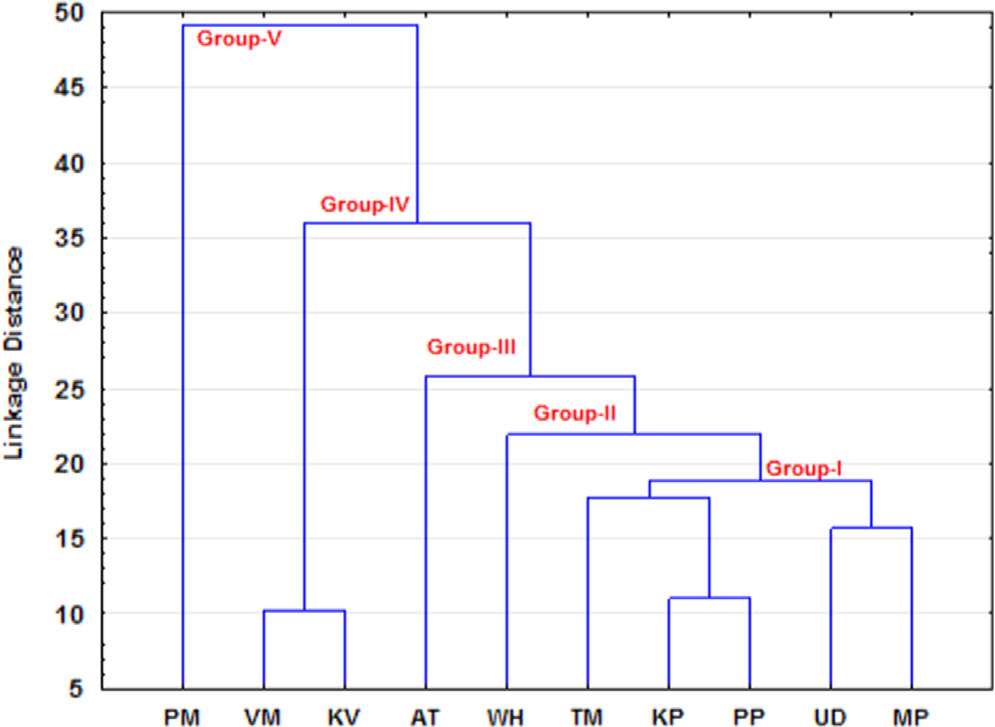
Cluster analysis of pottery samples.

A physical nature, period and image of collected ancient pottery samples are given in Table 1. The major and trace elemental concentration of ancient potteries are determined using the EDXRF technique and reported in Table 2. Factor loadings of major and trace elements of ancient potteries are given in Table 3 (STATISTICA (10.0 version) software). Fig. 1 shows the archeological excavation sites in the study area. Fig. 2, Fig. 3 represent the factor analysis and Fig. 4 shows the clustering analysis of major and trace elements.

## 2 Experimental design, materials and methods

### 2.1 Sample collections

Ten pottery samples collected from the ancient settlement sites in and around Arcot of Vellore District, Tamil Nadu, India (Fig. 1). The pottery samples are excavated in 6 m depth from surface of earth. After removal of surface layers, the pottery shreds were ground into fine powder using agate mortar and then stored in polythene bag [1]. These samples are cleaned and dried using hot air oven.

### 2.2 ED-XRF technique

One gram of the fine ground sample and 0.5 g of the boric acid (H_3_BO_3_) were mixed. The mixture was thoroughly ground and pressed into a pellet of 25 mm diameter using a 20 ton hydraulic press. The instrument EDXRF used for this analysis was a PW 4025 Minipal supplied by M/s Philips, Netherlands. The spectrometer was fitted with a side window X-ray tube (9 W) that had Rhodium as anode. The power specifications of the tube were 4–30 kV; 1 μA–1 mA. Removable sample changer of the instrument accommodated 12 samples at a time. Selection of filters, tube voltage, sample position and current were fully controlled by a Computer. Beam spot area (elliptical) for the instrument was 81.7 mm^2^. The instrument had the features of Multi Channel Analyzer (MCA) test, standardless determination and automatic gain correction. Gain correction was performed when the beam stop was in the reference position. Beam stop was fed with a reference sample (an alloy of aluminum and copper). Copper was used for gain correction and Al and Cu were used for calibrating instrument energy.

For elements Al, Ca, K, Fe, Si, Ti, V, Cu, Zn and Mn, the Kα lines and for Ba, La and Pb, the Lα lines were used to activate X-ray analysis. The region of interest for Co was 3.025–3.240 keV while that of Cd was 6.815–7.120 keV. For Cd and Co, the exposure time was 300 s. For elements Al, Ca, K, Fe, Si and Ti, the exposure time was 60 s. For elements Ba, Cu, Mn, Pb, V and Zn, time of exposure was 200 s.

### 2.3 Factor and cluster analysis

Factor and cluster analysis are used to identify pottery groups, which could be clearly differentiated from each other to establish an archeological classification. In this work, major and trace elemental data of pottery samples performed using STATISTICA (version10.0) software package for factor and cluster analysis [2]. Fig. 2, Fig. 3 (circles) show the concentration of elements reveals that grouping of ancient potteries.

## Figures and Tables

**Figure:**
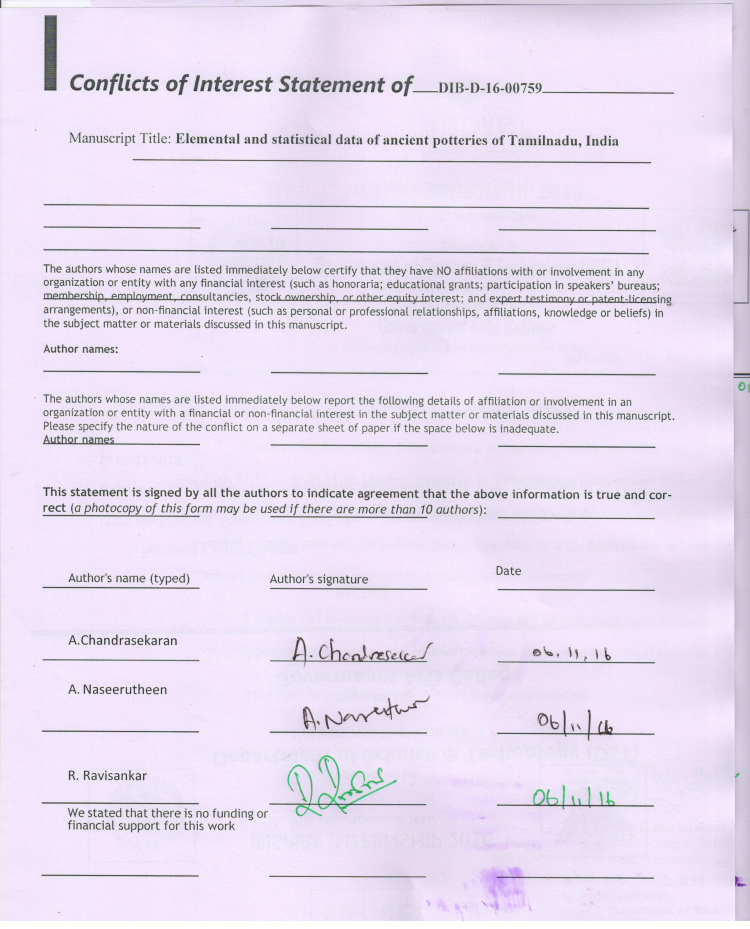
Supplementary material.

**Table 1:** Details of ancient potteries Arcot city, Tamil Nadu.

**S.No**	**Sampling sites**	**Sample ID**	**Inner color**	**Outer color**	**Approximate period**	**Pottery**
1	Melpadi	MP	Red	Red	900 AD	
2	Paiyampalli	PP	Red	Red	1200 AD	
3	Thirumani	TM	Brown	Red	600 AD	
4	Karivadu	KV	Black-Red	Dark-Black	900-1000 AD	
5	Arcoat	AT	Red (nice)	Red (nice)	200 BC	
6	Vallimalai	VM	Black-Red	Black	1200 AD	
7	Kavaripakkam	KP	Red	Red	1300 AD	
8	Panchapandavar mali	PM	Brown-Red	Brown	1500 AD	
9	Walajah	WH	Red	Red	900 AD	
10	Udayandiram	UD	Red-Black	Black-Red	200 BC	

**Table 2:** Elemental composition of ancient potteries in and around Arcot city, Tamil Nadu, India (* in percentage; #in ppm).

**Sampling sites/ element**	**Melpadi**	**Paiyam palli**	**Thirumani**	**Karivadu**	**Arcot**	**Vallimalai**	**Kavari pakkam**	**PanchaPan davarmali**	**Walajah**	**Udaya ndiram**
**Sample ID**	**MP**	**PP**	**TM**	**KV**	**AT**	**VM**	**KP**	**PM**	**WH**	**UD**
*SiO_**2**_	37.44	41.93	43	39.58	35.08	39.36	38.29	38.94	42.57	37.87
*Al_**2**_**O**_**3**_	6.61	7.75	7.18	6.24	7.56	6.8	6.42	7.37	6.99	8.31
*CaO	1.54	2.24	1.54	1.26	1.68	1.26	1.82	1.4	2.38	1.26
*Fe_**2**_**O**_**3**_	5.15	6.43	6.72	8.44	5.58	8.44	6.00	7.15	6.58	6.72
*K_**2**_**O**	0.96	1.2	1.81	1.33	1.57	1.69	1.45	2.05	2.17	2.77
*TiO_**2**_	0.67	0.83	0.67	0.83	0.67	0.7	0.68	0.58	0.5	0.58
**#Cu**	38.16	52.72	69.16	84.92	62.03	83.04	53.54	65.65	56.25	41.91
**#Zn**	130.82	131.44	131.18	153.17	152.93	161.15	126.6	106.87	127.12	139.2
**#Pb**	19.84	25.11	21.29	21.76	18.75	19.24	25.61	26.35	23.03	22.45
**#La**	23.4	25.38	26.34	33.45	28.09	31.65	25.95	24.81	22.33	22.09
**#Co**	15.52	21.67	22.27	31.24	18.5	30.64	19.95	24.42	21.06	21.5
**#V**	35.66	34.81	39.02	69.88	44.87	64.76	26.23	96.25	53.48	45.63
**#Cd**	0.21	0.41	0.63	0.51	0.58	0.55	0.49	0.77	0.53	1.09
**#Cr**	88.09	95.63	98.7	106.51	91.5	105.75	93.5	91.18	84.59	90.96

**Table 3:** Factor loading of elemental data.

Variables	Factor-1	Factor-2	Factor- 3
SiO_2_	0.256897	0.126844	−0.731877
Al_2_O_3_	−0.268324	−0.687692	0.047629
CaO	−0.488043	0.265709	−0.591899
Fe_2_O_3_	0.973803	−0.076487	−0.041148
K_2_O	0.105203	−0.944050	−0.092252
TiO_2_	0.336556	0.709427	0.185842
Cu	0.908103	0.203992	0.078597
Zn	0.372214	0.246369	0.766395
Pb	−0.013515	−0.164244	−0.818901
La	0.768064	0.463166	0.388711
Co	0.986833	0.021926	0.009225
V	0.658077	−0.370650	−0.145478
Cd	0.222314	−0.919210	0.073314
Cr	0.834465	0.353688	0.270792
% of variance explained	**40.79**	**23.07**	**14.80**
